# Investigation of serum amyloid a within animal species focusing on the 1-25 amino acid region

**DOI:** 10.1080/01652176.2023.2267605

**Published:** 2023-10-06

**Authors:** Natalie G. Horgan, Kendall B. E. Moore, Jessica S. Fortin

**Affiliations:** Department of Basic Medical Sciences, College of Veterinary Medicine, Purdue University, West Lafayette, IN, USA

**Keywords:** Animals, aggregation, amyloid-like fibrils, oligomers, serum amyloid A1, zoology

## Abstract

AA amyloidosis, characterized by the misfolding of serum amyloid A (SAA) protein, is the most common amyloid protein disorder across multiple species. SAA is a positive-acute phase protein synthesized by the liver in response to inflammation or stress, and it normally associates with high-density lipoprotein at its N-terminus. In this study, we focused on the 1-25 amino acid (aa) region of the complete 104 aa SAA sequence to examine the aggregation propensity of AA amyloid. A library comprising eight peptides from different species was assembled for analysis. To access the aggregation propensity of each peptide region, a bioinformatic study was conducted using the algorithm TANGO. Congo red (CR) binding assays, Thioflavin T (ThT) assays, and transmission electron microscopy (TEM) were utilized to evaluate whether the synthesized peptides formed amyloid-like fibrils. All synthetic SAA 1-25 congeners resulted in amyloid-like fibrils formation (per CR and/or ThT staining and TEM detection) at the exception of the ferret SAA1-25 fragment, which generated plaque-like materials by TEM. Ten residues were preserved among SAA 1-25 congeners resulting in amyloid-like fibrils, i.e. F6, E9, A10, G13, D16, M17, A20, Y21, D23, and M24. Amino acid residues highlighted by this study may have a role in increasing the propensity for amyloid-like fibril formation. This study put an emphasis on region 1-25 in the mechanism of SAA1 misfolding.

## 1. Introduction

AA Amyloid derives from the positive acute-phase protein serum amyloid A (SAA) (Real de Asua et al. 2014). The liver synthesizes SAA in response to inflammation, in which it can enhance the antioxidant potential of HDL cholesterol (Sato et al. 2016; Sun and Ye 2016). However, the misfolding of the N-terminus region of SAA can lead to amyloidosis, which is the accumulation of pathogenic misfolded protein amyloid in targeted tissue(s) (Westermark et al. 2015; Gaffney 2017). The aggregation and subsequent accumulation of amyloid occurs when these fibrillar peptides misfold into secondary structures, rich in ß-sheets (Real de Asua et al. 2014; Westermark et al. 2015). When a seeding-nucleation response occurs, fragmented SAA amyloid monomers recruit naïve, healthy SAA1 protein. It is through this prion-like transmission that β-sheet amyloid plaques accumulate, causing tissue damage. Furthermore, chronic inflammation causes high concentrations of serum SAA levels, leading to the misfolding of SAA. The deposition of SAA into the tissues of major organs results in extensive damage and the possibility of organ failure.

AA Amyloidosis is the most common type of amyloid disease reported in both humans and animals (Gaffney 2017). AA Amyloidosis has a critical impact on zoo and wildlife species due to cross-species transmission (CST). CST allows for this disease to spread throughout the zoo and wildlife communities, both fecal-orally and parenterally (Page et al. 2005). The amount of fibril deposition is dependent on the quantity of fibril, route of admission, and the occupancy of tissue homogenate (Cui et al. 2002). The transmission of disease between species can be managed through the minimization of stress; however, stress cannot be eliminated entirely.

AA amyloidosis is thought to be a secondary reactive amyloidosis in response to primary diseases that cause inflammation, including infections, neoplasms, and immune disorders (Lu et al. 2014). As such, clinical appearance varies among species. In animals, deposition of AA fibrils and pathogenesis have been detected in several organs such as the kidney, spleen, liver, arterial walls, and gastrointestinal tract. Of these, the kidneys usually contain the highest accumulation of amyloid fibril deposition, with the most common initial clinical manifestation being renal dysfunction (Lachmann et al. 2007). Currently, it is not possible to predict the spreading of AA fibrils due to differing distribution, clinical signs, and pathological findings across species.

The eight species selected for this study include the human (*Homo sapiens*), common bottlenose dolphin (*Tursiops truncatus*), donkey (*Equus asinus*), rhesus monkey (*Macaca mulatta*), alpine ibex (*Capra ibex*), lesser-Egyptian jerboa (*Jaculus jaculus*), domestic ferret (*Mustela putorius furo*), and chamois (*Rupicapra rupicapra*). Naturally occurring systemic amyloidosis due to misfolded SAA1 has been reported in humans (*Homo sapiens*) (Gaffney 2017) and the common bottlenose dolphin (*Tursiops truncatus*) (Cowan 1995). The following additional species have been reported cases of systemic amyloidosis (presumably from SAA misfolding): rhesus monkey (*Macaca mulatta*) and the chamois (*Rupicapra rupicap*). Based on our literature search (from 2010-2023), there are no case reports found in the donkey (*Equus asinus*) (although cases have been reported in horses), the alpine ibex (*Capra ibex*), the Lesser-Egyptian jerboa (*Jaculus jaculus*), or the domestic ferret (*Mustela putorius furo*) (although systemic amyloidosis occurred in black-footed ferret (*Mustela nigripes*)).

During analysis, our study was expanded to include ten additional species, including the chicken (*Gallus gallus*), canine (*Canis lupus familiaris*), Amur tiger (*Panthera tigris altaica*), zebra finch (*Taeniopygia guttata*), budgerigar (*Melopsittacus undulatus*), turkey (*Meleagris gallopavo*), rainbow trout (*Oncorhynchus mykiss*), Chinese soft-shelled turtle (*Pelodiscus sinensis*), mallard (*Anas platyrhynchos*), and Tasmanian devil (*Sarcophilus harrisii*). Further, our analysis included the Japanese quail (*Coturnix japonica*), which shares its 1-25 aa sequence with the turkey. These species were chosen based on their RAY alignment, specifically where the “RAY” amino acids align consistently at positions 19-21 on the SAA 1-25 aachain across all selected species. However, their signal peptide sequences may exhibit a minor variation in the amino acid length. Despite this discrepancy, their aggregation scores remain largely unchanged, which is why they have been included as supplementary data.

To identify specific residue regions that contribute to the aggregation of SAA and point-mutation variances, *in silico* analysis was utilized. The library of eight different peptide sequences was arranged using BioEdit and the aggregation propensity was calculated using the TANGO algorithm (Fernandez-Escamilla et al. 2004; Linding et al. 2004; Rousseau et al. 2006). The bioinformatic study was supported through a series of *in vivo* assays, including Thioflavin (ThT) T binding assay, transmission electron microscopy (TEM), and Congo red (CR) binding assays to identify formation and quantification of developed amyloid-like fibrils.

## 2. Material and methods

### 2.1. Chemicals and peptides

The peptides were purchased from GenScript USA Inc (Piscataway, NJ). The peptides were synthesized (commercial quality) and received in a crude form, with purity levels ranging between 50% and 70%. The Congo red, hexafluoroisopropanol, and thioflavin T were obtained from Alfa Aesar (Ward Hill, MA).

**Figure 1.**
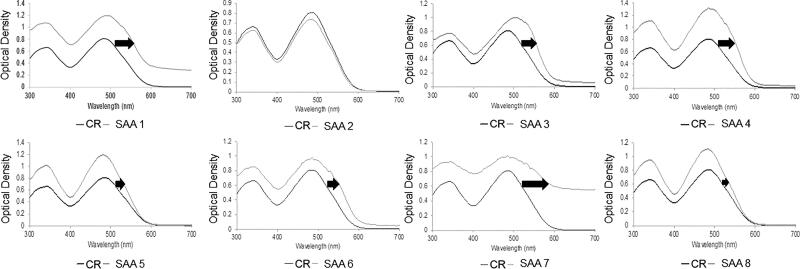
Representative visible spectra obtained from Congo red (CR) binding assays of the eight serum amyloid A1 synthetic peptides of the region 1-25 at 500 µM. Optical density (OD) data were plotted as function of wavelength (nm). The change in optical density (higher and right shift) is indicative of CR binding to β-plated sheets. Optical density measurements were recorded at 25 °C in 25 mM Tris buffer (pH 8) and 50% hexafluoroisopropanol (HFIP) after 5 days of incubation. ID# 1: human; ID# 2: common bottlenose dolphin; ID# 3: donkey; ID# 4: rhesus monkey; ID# 5: alpine ibex; ID# 6: Lesser-Egyptian jerboa; ID# 7: domestic ferret; ID# 8: chamois.

### 2.2. Congo red (CR) binding assays

A CR stock solution (0.0035 g/ml, 5 pM) was prepared in phosphate buffered saline at pH 7.4 and filtered through a 0.2 µm syringe filter prior to use. The Congo red (CR) binding assays were performed in a 96 wells plate. Each well contained 25 µL of a signal peptide stock solution at 1 mM with 50% HFIP (final peptide concentration ∼500 µM), 25 µL of 50 mM Tris buffer (pH 8), and 5 µL of CR stock solution (final concentration of 0.5 pM). The 96 wells plate was incubated for 5 days at room temperature to allow amyloid-like fibril formation (without agitation). The plate was read at 300 - 700 nm to monitor CR binding using a Synergy HT multi-mode microplate reader (BioTek, Winooski, VT). The absorbance spectrum of Tris buffer was subtracted for each peptide and negative control (non-bond CR).

### 2.3. In silico analysis of aggregation

SAA1 peptide sequences were obtained from the NCBI database. The 1-25 aa section were subjected to the same analysis. Using the BioEdit software, the sequences were aligned to identify the variations present among the peptide fragments. The aggregation propensity (aggregation score) was obtained using the TANGO algorithm (Fernandez-Escamilla et al. 2004; Linding et al. 2004; Rousseau et al. 2006).

### 2.4. Reference database and accession numbers

Species were cross-referenced in both NCBI and UniProt databases, each assigned a unique accession number. In NCBI, the following accession numbers were recorded: Human (*Homo sapiens*): AAA60297, Common bottlenose dolphin (*Tursiops truncatus*): BAM48873, Donkey (*Equus asinus*): XP_044608461, Rhesus monkey (*Macaca mulatta*): EHH23058, Alpine ibex (*Capra ibex*): ACH73010, Lesser-Egyptian jerboa (*Jaculus jaculus*): XP_045001623, Domestic ferret (*Mustela putorius furo*): NP_001297109, and Chamois (*Rupicapra rupicapra*): CBJ23335. In UniProt, the corresponding accession numbers are as follows: Human (*Homo sapiens*): P0DJI8, Common bottlenose dolphin (*Tursiops truncatus*): K0J8C4, Donkey (*Equus asinus*): A0A8C4LEB1, Rhesus monkey (*Macaca mulatta*): G7NDT0, Alpine ibex (*Capra ibex*): B6D982, Lesser-Egyptian jerboa *(Jaculus jaculus*): A0A8C5KXT5, Domestic ferret (*Mustela putorius furo*): K0J5R8, and Chamois (*Rupicapra rupicapra*): E1UYU4.

### 2.5. Thioflavin T (ThT) fluorescence experiment with different SAA 1-25 peptides

ThT fluorescence assays were conducted to detect fibril formation in the fragmented peptides. A 1 mM protein solution was prepared using 100% HFIP for each species. In a non-treated 96-well microplate with a transparent bottom, 50 µL of the protein solution (final concentration 500 µM) and 50 µL of Tris-hydroxymethyl-aminomethane (Tris Buffer, pH 8) (final concentration at 25 mM) were dispensed. Each peptide was replicated into three repeated wells. ThT was added last at a concentration of 100 µM. To establish the background signal, a negative control was prepared and contained Tris buffer and ThT. The Synergy HT multi-mode microplate reader, set at an excitation wavelength of 440 nm and an emission wavelength of 485 nm, was used to measure the ThT fluorescence after the plate was sealed. Prior to each reading, the plate reader was slowly shaken for ten seconds. The fluorescence measurements were recorded every hour for duration of five days, and the procedure was repeated to ensure consistency. Statistical analysis of results involved calculating the average of the last five data points after subtracting the background signal.

### 2.6. Transmission electron microscopy (TEM)

SAA1 peptides, specifically amino acids 1-25, were placed in a 1.5 mL microcentrifuge test tube at a concentration of 500 µM. The peptide solution was prepared using 1:1 volume ratio of HFIP and 50 mM Tris buffer with fragment peptide at 1 mM concentration (pH 8), which resulted in a final concentration of 50% HFIP and 25 mM Tris buffer with 500 µM of peptide (pH 8). Total volume was of 75 µL. To mitigate potential concentration changes, the test tubes were sealed with parafilm. This parafilm barrier was intended to minimize HFIP evaporation. We have monitored the loss of volume for an incubation of about seven days at 37 °C using three 1.5 mL microcentrifuge test tubes containing same solution. Incubations were done in a cell culture incubator with moisture atmosphere and 5% CO_2_. We confirmed that there was still a sufficient amount of HFIP present in the microcentrifuge test tubes throughout the entire incubation period as the percentage of volume lost ranged from 0% to 1.7%. Following preparation, the test tubes containing the peptide solution were incubated at 37 °C without any stirring or agitation for seven days (using cell culture incubator). After the incubation period was complete, each tube was centrifuged at 14,000 rpm for ten minutes. The supernatant was carefully removed, discarding the HFIP, and replaced with the addition of 100 µL of phosphate-buffered saline (pH 7.4). Approximately 10 µL of the peptide solution was deposited onto the 400-mesh Formvar-carbon-coated copper grid (Electron Microscopy Sciences, Hatfield, PA). The grids were incubated for one minute at room temperature before being washed three times with distilled water. The grid was blotted dry with filter paper and then air-dried. Approximately 1–2 µL uranyl acetate was added to the grid for 1 min. Excess fluid was blotted dry again with filter paper and left to air-dry. To complete this process, transmission electron microscopy was employed to capture images of the grids using an accelerating voltage of 100 kV and magnification of 40k (JEOL 1400 Flash, Japan).

## 3. Results

### 3.1. Aggregation score of the 1-25 amino acid region of the SAA1 peptide in different species

Upon examination of the human SAA1 peptide sequence, it was observed that the 1-25 aa region exhibited a tendency for aggregation (Haines et al. 2022). To expand the investigation to other species, sequences of the SAA1 peptide from eight different species, with a specific focus on the 1-25 aa region, were obtained. Point mutations in comparison to the human sequence, which served as the control, were highlighted in red (Table 1). To further explore this region across species, an aggregation score was calculated using the TANGO computer algorithm. The analysis revealed a range of aggregation scores from 0 to 448 among the eight species. The common bottlenose dolphin-associated peptide (#2) exhibited the lowest aggregation score, while the donkey-associated peptide (#3) exhibited the highest aggregation score.

**Table 1.** 1-25 Amino acid peptide sequence library of the serum amyloid A1 (SAA1) in eight species.

ID no.	Species	1	2	3	4	5	6	7	8	9	10	11	12	13	14	15	16	17	18	19	20	21	22	23	24	25	AGG
1	Human (*Homo sapiens*)	R	S	F	F	S	F	L	G	E	A	F	D	G	A	R	D	M	W	R	A	Y	S	D	M	R	376
2	Common bottlenose dolphin (*Tursiops truncatus*)	Q	R	W	G	T	F	L	K	E	A	G	Q	G	A	K	D	M	W	R	A	Y	S	D	M	R	3
3	Donkey (*Equus asinus*)	R	E	W	F	T	F	L	K	E	A	G	Q	G	A	K	D	M	W	R	A	Y	S	D	M	R	448
4	Rhesus Monkey (*Mucaca mulatta*)	R	S	W	F	S	F	L	G	E	A	Y	D	G	A	R	D	M	W	R	A	Y	S	D	M	K	238
5	Alpine ibex (*Capra ibex*)	Q	G	W	G	T	F	L	R	E	A	G	Q	G	A	K	D	M	W	R	A	Y	K	D	M	K	11
6	Lesser-Egyptian jerboa (*Jaculus jaculus*)	Q	G	W	Y	Q	F	M	K	E	A	G	Q	G	A	R	D	M	W	H	A	Y	S	D	M	K	14
7	Domestic ferret (*Mustela putorius furo*)	Q	R	W	L	G	F	L	K	E	A	G	Q	G	A	R	D	M	Y	R	A	Y	S	D	M	R	26
8	Chamois (*Rupicapra rupicapra*)	Q	G	W	G	T	F	L	R	E	A	G	Q	G	T	K	D	M	W	R	A	Y	R	D	M	K	11

The sequences were obtained *via* NCBI, and the aggregation score (AGG) was predicted by TANGO.

### 3.2. Congo red (CR) binding assays with the fractioned peptides exhibiting fibrillar β-aggregates

CR dye binds to peptides that form fibrillar β-aggregates, however not all fibrils are Congophilic. CR dye may bind at different sites with different binding affinity to wide variety of fibrils in comparison with ThT (Gade Malmos et al. 2017; Frieg et al. 2020). This CR binding assay offer a different mean to monitor amyloid-like fibril formation. Peptides with CR dye were incubated for 5 days at room temperature at 500 µM. Negative control consisted of the CR solution alone. Visible spectral data (300 − 700 nm) was acquired to capture the characteristic shift in absorbance maximum (485–501 nm). The difference spectra (free CR vs bound CR) are indicative of the amyloid-like beta-pleated sheet structure. For the CR binding assays, the peptides were processed at a concentration of 500 µM with 50% HFIP. Curves exhibiting high and right shift are considered positive for CR binding (Figure 1). Almost all SAA 1-25 fragment peptides were CR positive at a concentration of 500 µM except for common bottlenose dolphin SAA 1-25 fragment (Figure 1).

### 3.3. Monitoring fibril formation of various SAA 1-25 congeners via Thioflavin T (ThT) fluorescence

**Figure 2.**
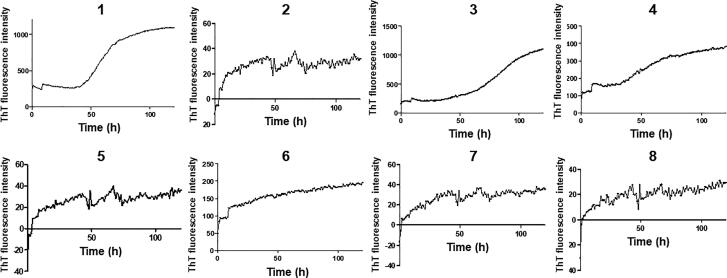
Comparison of Thioflavin T (ThT) fluorescence intensity obtained at the end of fibrilization kinetics for synthetic SAA 1-25 fragments resourced from amino acid sequences of different animal species. The experiments were conducted using a concentration of 500 μM in 25 mM Tris buffer (pH 8) and 50% hexafluoroisopropanol (HFIP). Fragment peptides were monitored at 37 °C for 120 h. Average represents experimental triplicate. Fluorescence background signal was subtracted. Each numerical value in the figure corresponds with the respective organism as presented in Table 1. Specifically, ID# 1: human; ID# 2: common bottlenose dolphin; ID# 3: donkey; ID# 4: rhesus monkey; ID# 5: alpine ibex; ID# 6: Lesser-Egyptian jerboa; ID# 7: domestic ferret; ID# 8: chamois.

**Figure 3.**
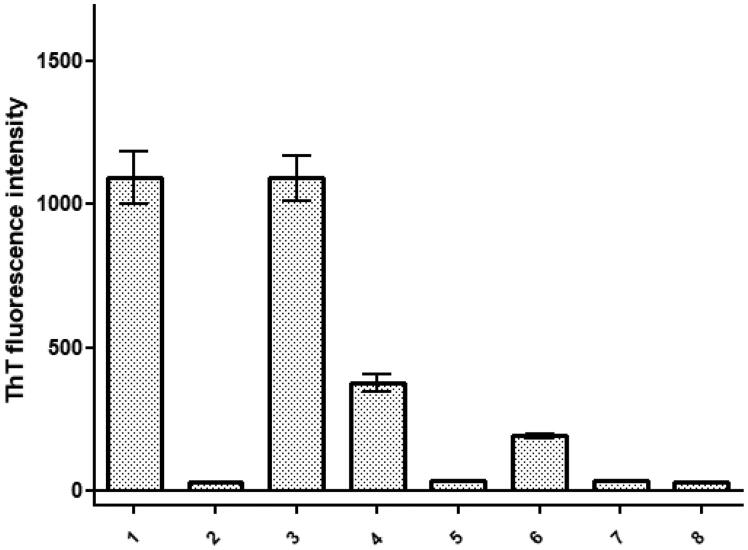
Comparison of the Thioflavin T (ThT) fluorescence intensity. Graphs represent each species’ fibrilization intensity over 120 h (five days) in incubation at 37 °C. Three samples ran simultaneously were then averaged subtracting the background intensity from each prior to creating the graph. Error bars represent SEM. ID# 1: human; ID# 2: common bottlenose dolphin; ID# 3: donkey; ID# 4: rhesus monkey; ID# 5: alpine ibex; ID# 6: Lesser-Egyptian jerboa; ID# 7: domestic ferret; ID# 8: chamois.

Among the eight peptides evaluated, all species exhibited positive evidence of aggregation, as indicated by fluorescence readings above the background level (previously subtracted). The kinetics of the fibril formation for each fragment peptide are showed in Figure 2. The common bottlenose dolphin, alpine ibex, domestic ferret, and chamois peptides SAA 1-25 fragment peptides displayed very low fluorescence intensity. Conversely, the human peptide and the donkey peptide exhibited significantly increased fluorescence, indicating a high level of aggregation of the serum amyloid A peptide in these species. The ThT fluorescence visualization in Figure 3 demonstrated the aggregation intensity over the course of 120 h. The human, donkey, and rhesus monkey peptides displayed a sigmoidal increase in aggregation intensity. In contrast, the peptide from the Lesser-Egyptian jerboa resulted in a steady and rapid increase in aggregation intensity. Finally, the peptides from the common bottlenose dolphin, domestic ferret, and chamois showed consistent intensities at various levels throughout the 120-h timeframe.

### 3.4. Visualization of the different SAA 1-25 peptides by transmission electron microscopy (TEM)

**Figure 4.**
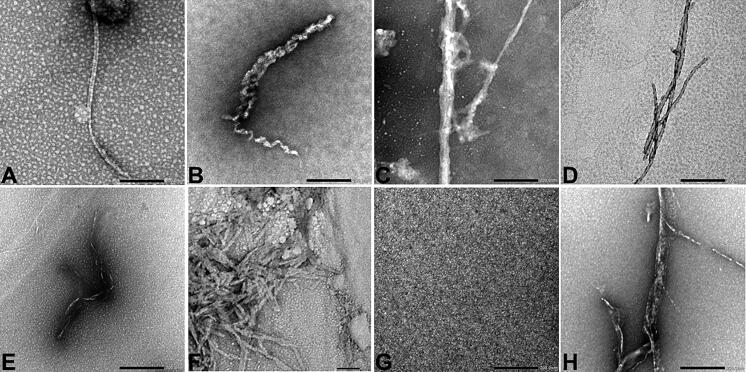
Transmission electron microscopy (TEM) was used to observe the different synthetic SAA fragment peptides solubilized at 500 µM in 25 mM Tris buffer (pH 8) and 50% hexafluoroisopropanol (HFIP), and subsequently incubated at 37 °C for seven days, at a magnification of 40K. Notation a corresponding to the human SAA1 peptide. Notation B corresponding to the common bottlenose dolphin SAA1 peptide. Notation C corresponding to the donkey SAA1 peptide. Notation D corresponding to the rhesus monkey SAA1 peptide. Notation E corresponding to the alpine ibex SAA1 peptide. Notation F corresponding to the Lesser-Egyptian jerboa SAA1 peptide. Notation G corresponding to the domestic ferret SAA1 peptide. Notation H corresponding to the chamois SAA1 peptide. Scale bar = 200 nm.

**Figure 5.**
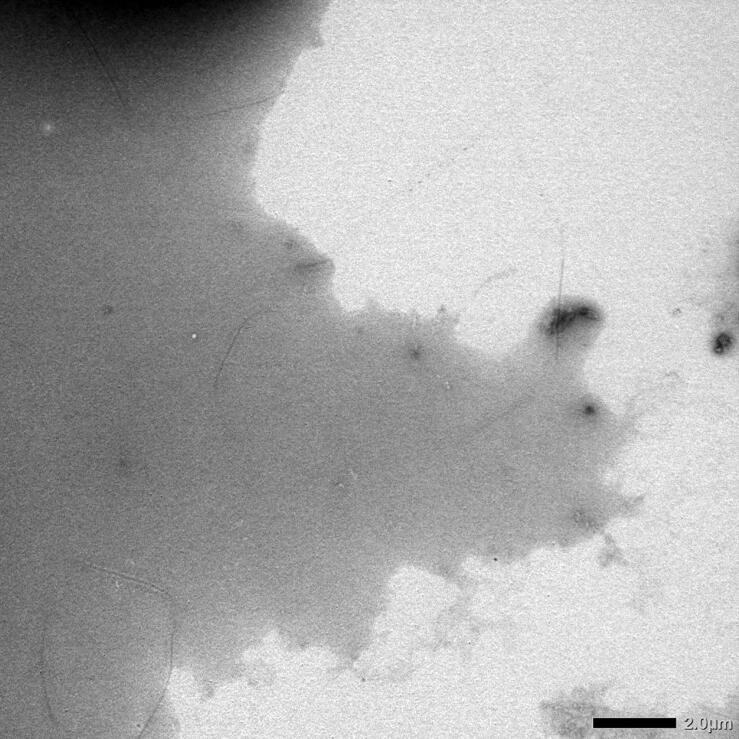
Photomicrograph of the domestic ferret SAA1 peptide taken at low magnification (2000) by transmission electron microscopy. Experimental conditions are identical as in Figure 4.

TEM analysis indicated the presence of amyloid formation in all but one of the peptides (Figure 4). Fibrillar structures were observed in SAA 1-25 fragment peptides inspired from human, common bottlenose dolphin, donkey, rhesus monkey, alpine ibex, Lesser-Egyptian jerboa, and chamois. The domestic ferret SAA 1-25 fragment peptide did not show amyloid-like fibrils at high magnification, but rather amyloid-like plaque materials at low magnification (Figure 5).

## 4. Discussion

Serum amyloid A has the propensity to aggregate in a variety of species. Amyloid-like fibril formation, also known as amyloidogenesis, results when the N-terminus portion of the SAA1 protein converts from an alpha helix to ß-sheet structure. The purpose of this study was to assess the 1-25 amino acid sequence of the SAA peptide in a variety of species. This region was previously deemed an aggregation hotspot and seeding factor of the SAA peptide within humans (Haines et al. 2022). In addition, our previous work on the SAA1 human protein indicated that the region 1-25 is highly prone to aggregate (Haines et al. 2022). Bernhardt et al. showcased that the SAA 1-13 aa sequence is prone to disaggregate into ß-sheets increasing the probability of forming aggregates (Bernhardt et al. 2016). Another study from Skibiszewska and colleagues reported that synthesized segments of both SAA 1-12 aa and 1-27 aa have high capacity to aggregate and form oligomers (Skibiszewska et al. 2020). Finally, a team in Japan reports that the SAA N-terminus 2-15 aa region promotes amyloid fibril formation upon investigation of the fragment isolated from human patients postmortem (Shintani-Domoto et al. 2022).

Across the eight SAA 1-25 congeners included in our study, ten residues are preserved: F6, E9, A10, G13, D16, M17, A20, Y21, D23, and M24. The six latter residues are preserved among the ten species included in the Supplemental Data section, as well. These residues may increase propensity for fibril formation since they are present in all species, which each depicted evidence of fibrils upon experimentation. W18 is another residue of note; this is conserved across seven of the eight species, with the ferret being the exception. The ferret depicted the least amount of fibril formation, potentially signifying that the deviation in residue 18 is indicative of restrained fibril growth.

An interesting point of discussion is the differences observed in *ex vivo* amyloid structure compared to those *in vitro*. As discussed by Fandrich et al. *ex vivo* fibrils contain more beta-sheets when visualized *via* cryo-EM. The examination of structural differences in fibrils, both *ex vivo* and *in vitro*, among these eight species, could provide valuable insights into the factors that impact the pathogenicity of fibrils isolated from patients. Using cryo-EM to visualize fibrillar nature has been conducted on feline, murine, and human amyloid fibrils (Liberta et al. 2019; Schulte et al. 2022). Our literature search yielded no evidence of previous studies using cryo-EM to visualize amyloidosis fibrils of other species. Performing cryo-EM on fibrils sourced from our eight chosen species would expand the knowledge of amyloid structure.

Another potential investigation could include the use of heparin to treat SAA peptides in order to induce the formation of amyloid-like fibrils. Due to the abundance of lysine and arginine in the amino acid sequences, heparin is promising for promoting amyloid-like fibril growth and nucleation. Heparin’s positively charged amino acids enable it to facilitate the nucleation process by binding to proteins and promoting the adoption of the β-sheet-rich conformation essential for amyloid formation. This phenomenon, as demonstrated with the basic parathyroid hormone (PTH1-84), involves heparin inducing a structural shift in PTH1-84, increasing α-helical content and decreasing β-strand content, thereby enhancing its inclination toward amyloid-prone conformations (Lauth et al. 2022). Such exploration into heparin’s role in fibril formation would allow for deeper studies into the structure of fibrils.

The aggregation of SAA has been identified in non-human species (Murakami et al. 2014; 2015). Cowan was among the first to report amyloidosis in the kidneys and blood vessels of dolphins (Cowan 1995). AA amyloidosis is well-published in the mouse, with reports of aggregates in the liver, spleen, and kidneys of mice (Simons et al. 2013; Vahdat Shariat Panahi et al. 2019; Lin et al. 2021). Food animals (predominantly goats (Lin et al. 2021), bovine (Husebekk et al. 1988), and poultry (Higuchi 2013)) are not immune to SAA accumulation, either, posing a human health risk upon consumption of aggregates in tissue (Rising et al. 2021).

The SAA 1-25 aa fragment depicts a high potential to aggregate amongst various species, as demonstrated through our experimentation. Such adds to the pool of literature supporting the propensity of the 1-25 aa region to aggregate. Investigating misfolded SAA protein and subsequent amyloidosis proves important due to the wide range of species affected and the potential for interspecies transmission. AA amyloidosis has been found to be transmitted between species both naturally and when artificially induced. Murakami et al. reviewed transmission of amyloid fibrils *via* oral administration (Murakami et al. 2015). They summarized that several species, including the mouse, cheetah, cat, mink, human, and cow, developed amyloidosis when administered SAA1 of either same-species or different species origin (Murakami et al. 2015). In a subsequent review, Murakami et al. elaborates upon the lack of literature regarding human susceptibility to developing amyloidosis from consumption or interaction with contaminated products, including meat, water, and the environment (Murakami et al. 2014). These concerns are not to be disregarded; one study reports a 5% occurrence rate of amyloidosis in one group of 302 slaughtered cattle (Tojo et al. 2005). Due to the potential for transmission across and within species, there is reason to be worried for both human public health and the health of species with whom we frequently interact. A greater understanding of SAA1 and subsequent amyloidosis will open the door to the development of therapeutics to ameliorate amyloid fibrils or slow disease progression.

## Supplementary Material

Supplemental Material
