# Supplementary material for: Investigation of serum amyloid a within animal species focusing on the 1-25 amino acid region
Source: Vet Q. 2023 Oct 6;43(1):1–8. doi: 10.1080/01652176.2023.2267605 (PMC10614707; doi:10.1080/01652176.2023.2267605)
Supplement: Supplemental Material [file TVEQ_A_2267605_SM1944.docx]

**Investigation of serum amyloid A within animal species focusing on the 1-25 amino acid region**

Natalie G. Horgan^1°^, Kendall B. E. Moore^1°^, Jessica S. Fortin^1*^

^1^Department of Basic Medical Sciences, College of Veterinary Medicine, Purdue University, 625 Harrison Street, West Lafayette, Indiana, USA: Department of Pathobiology and Diagnostic Investigation, College of Veterinary Medicine, 47907

^°^Contributed equally

*Corresponding author

**Supplemental document**

## During analysis, our study was expanded to include ten additional species, including the chicken (*Gallus gallus*), canine (*Canis lupus familiaris*), Amur tiger (*Panthera tigris altaica*), zebra finch (*Taeniopygia guttata*), budgerigar (*Melopsittacus undulatus*), turkey (*Meleagris gallopavo*), rainbow trout (*Oncorhynchus mykiss*), Chinese soft-shelled turtle (*Pelodiscus sinensis*), mallard (*Anas platyrhynchos*), and Tasmanian devil (*Sarcophilus harrisii*). Further, our analysis included the Japanese quail (*Coturnix japonica*), which shares its 1-25 aa sequence with the turkey. These species were chosen based on their RAY alignment (i.e. alignment via aa residues 19 to 21); however, their signal peptide sequences exhibit a minor variation in amino acid positioning. Despite this discrepancy, their aggregation scores remain largely unchanged, which is why they have been included as supplementary data.

For the chicken, budgerigar, turkey/Japanese quail, Chinese soft-shelled turtle, mallard, and Tasmanian devil the signal peptide consists of 18 amino acids. The sequence indicated start at amino acid 5 after the cleavage of signal peptide region. Due to the RAY alignment, fragment peptides ID #9, 13, 14, 16-18 do not contain the expected N-terminal region.

For the canine, Amur tiger, and rainbow trout the signal peptide consists of 18 amino acids. Due to the RAY alignment, the fragment peptides ID #10, 11, 15 include the last amino acid of the signal peptide and does not start at the expected N-terminal region.

For the zebra finch, the signal peptide consists of 18 amino acids. The sequence indicated start at amino acid 10 after the cleavage of signal peptide region. Due to the RAY alignment, fragment peptide ID #12 does not contain the expected N-terminal region.

Naturally occurring systemic amyloidosis due to misfolded SAA1 has been reported in the chicken (*Gallus gallus*) (Murakami et al. 2013), the canine (*Canis lupus familiaris*) (DiBartola et al. 1990), the Amur tiger (*Panthera tigris altaica*) (Schulze et al. 1998), the zebra finch (*Taeniopygia guttata*) (Shientag et al. 2019; Shientag et al. 2016), and the Japanese quail (*Coturnix japonica*) (Nakayama et al. 2017). In addition, systemic amyloidosis associated with SAA has been frequently reported with ducks (Anatidae) (Shientag et al. 2016; Woo et al. 2017). Concerning the budgerigar (*Melopsittacus undulatus*), several cases of systemic amyloidosis have been reported in Psittaciformes (Nemeth et al. 2016). Based on our knowledge, there are no case reports found during years 2010-2023 in the rainbow trout (*Oncorhynchus mykiss*), the Chinese soft-shelled turtle (*Pelodiscus sinensis*), or the Tasmanian devil (*Sarcophilus harrisii*).

Species were cross-referenced in both NCBI and UniProt databases, each assigned a unique accession number. In NCBI, the following accession numbers were recorded: chicken (*Gallus gallus*) - NP_001366205, canine (*Canis lupus familiaris*) - XP_038285369, Amur tiger (*Panthera tigris altaica*) - AGU01750, zebra finch (*Taeniopygia guttata*) - XP_030129721, budgerigar (*Melopsittacus undulatus*) - XP_030908475, turkey (*Meleagris gallopavo*) - XP_010709376, rainbow trout (*Oncorhynchus mykiss*) - NP_001117908, Chinese soft-shelled turtle (*Pelodiscus sinensis*) - NP_001273858, mallard (*Anas platyrhynchos*) - NP_001297733, Tasmanian devil (*Sarcophilus harrisii*) - XP_003773620, and the Japanese quail (*Coturnix japonica*) - XP_015719668. In UniProt, the corresponding accession numbers are as follows: chicken (*Gallus gallus*) - F1NW65, canine (*Canis lupus familiaris*) - P19708, Amur tiger (*Panthera tigris altaica*) - T1W3H8, zebra finch (*Taeniopygia guttata*) - A0A674H7N9, budgerigar (*Melopsittacus undulatus*) - A0A8C6IMJ2, turkey (*Meleagris gallopavo*) - A0A803XXC3, rainbow trout (*Oncorhynchus mykiss*) - A6PZ09, Chinese soft-shelled turtle (*Pelodiscus sinensis*) - F4ZM51, mallard (*Anas platyrhynchos*) - P02740, Tasmanian devil (*Sarcophilus harrisii*) - G3X1D9, and the Japanese quail (*Coturnix japonica*) - A0A8C2TJQ9.

**Supplemental Table 1.** Additional library of fragment peptides of 25 amino acids. Peptide sequences were aligned with the human SAA RAY region (i.e. alignment via amino acids 19 to 21), which implies that some of the peptides do not include the N-terminal region or include a portion of the signal region. Aggregation scores were generated via Tango Algorithm. Amino acid divergence from the human SAA 1-25 region is indicated in red.

| **ID #** | **Species** | **1** | **2** | **3** | **4** | **5** | **6** | **7** | **8** | **9** | **10** | **11** | **12** | **13** | **14** | **15** | **16** | **17** | **18** | **19** | **20** | **21** | **22** | **23** | **24** | **25** | **AGG** |
| --- | --- | --- | --- | --- | --- | --- | --- | --- | --- | --- | --- | --- | --- | --- | --- | --- | --- | --- | --- | --- | --- | --- | --- | --- | --- | --- | --- |
| **1** | **Human (*Homo sapiens*)** | R | S | F | F | S | F | L | G | E | A | F | D | G | A | R | D | M | W | R | A | Y | S | D | M | R | 376 |
| **9** | **Chicken (*Gallus gallus*)** | S | S | G | I | K | F | V | R | D | A | A | G | G | A | R | D | M | W | R | A | Y | R | D | M | R | 0 |
| **10** | **Canine (*Canis lupus familiaris*)** | G | Q | W | Y | S | F | V | S | E | A | A | Q | G | A | W | D | M | W | R | A | Y | S | D | M | R | 287 |
| **11** | **Amur Tiger (*Panthera tigris altaica*)** | S | R | W | Y | L | F | L | G | E | G | A | Q | G | A | W | D | M | W | R | A | Y | F | D | M | R | 478 |
| **12** | **Zebra Finch (*Taeniopygia guttata*)** | I | R | A | G | Q | F | V | R | D | A | A | G | G | A | R | D | M | Y | R | A | Y | K | D | M | R | 0 |
| **13** | **Budgerigar (*Melopsittacus undulatus*)** | A | R | G | G | R | F | V | W | D | A | L | G | G | A | K | D | M | Y | R | A | Y | Q | D | M | R | 1 |
| **14** | **Turkey (*Meleagris gallopavo*) and Japanese quail (*Coturnix japonica*)** | S | S | G | F | K | F | V | R | D | A | A | G | G | A | R | D | M | W | R | A | Y | R | D | M | R | 0 |
| **15** | **Rainbow trout (*Oncorhynchus mykiss*)** | A | Q | W | Y | R | F | P | G | E | A | A | Q | G | A | K | D | M | W | R | A | Y | G | D | M | K | 0 |
| **16** | **Chinese soft-shelled turtle (*Pelodiscus sinensis*)** | T | N | A | G | A | F | I | R | D | A | Y | R | G | A | G | D | M | W | R | A | Y | D | D | M | R | 3 |
| **17** | **Mallard (*Anas platyrhynchos*)** | T | R | G | G | R | F | V | L | D | A | A | G | G | A | W | D | M | L | R | A | Y | R | D | M | R | 0 |
| **18** | **Tasmanian devil (*Sarcophilus harrisii*)** | T | D | S | A | R | F | L | K | E | A | G | Q | G | A | G | D | M | W | R | A | Y | R | D | M | R | 0 |

## **Results**

## SAA 1-25 fragment peptides ID# 9, 10, 11, 12, 15, 16, 17, 18 resulted in increased ThT fluorescence intensity in contrast to ID# 13 and 14 (**Supplemental Figure 1**). Sigmoidal time-course curves of amyloid-like fibril formation were obtained with most SAA 1-25 fragment peptides at the exception of SAA 1-25 fragment ID# 13 and 14 which were rather flat (**Supplemental Figure 2**). Fibrillar structures were observed for SAA 1-25 fragment peptides ID# 9-13, and 15-18 (**Supplemental Figure 3**). SAA fragment peptide ID# 14 (turkey and Japanese quail) resulted in spherical structures by TEM.


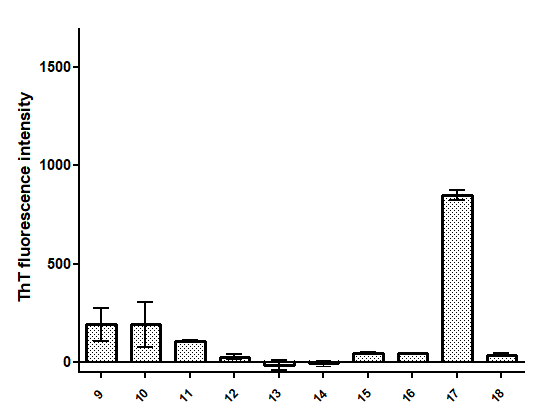


**Supplemental Figure 1.** Comparison of Thioflavin T (ThT) fluorescence intensity obtained at the end of fibrilization kinetics for various species of SAA1 protein. The experiments were conducted using a concentration of 500 μM in 25 mM Tris buffer (pH 8) and 50% hexafluoroisopropanol (HFIP). Three samples of the species peptide were monitored at 37 °C for 120 hours. Fluorescence intensity of each sample fluorescence was subtracted by background signal, and the differences between the three averages is represented by the bar. Each numerical value in the figure corresponds to synthetic SAA 1-25 fragment peptide as the following: ID# 9: chicken; ID# 10: canine; ID# 11: Amur tiger; ID# 12: Zebra finch; ID# 13: budgerigar; ID# 14: turkey and Japanese quail; ID# 15: rainbow trout; ID# 16: Chinese soft-shelled turtle; ID# 17: mallard; ID# 18: Tasmanian devil.


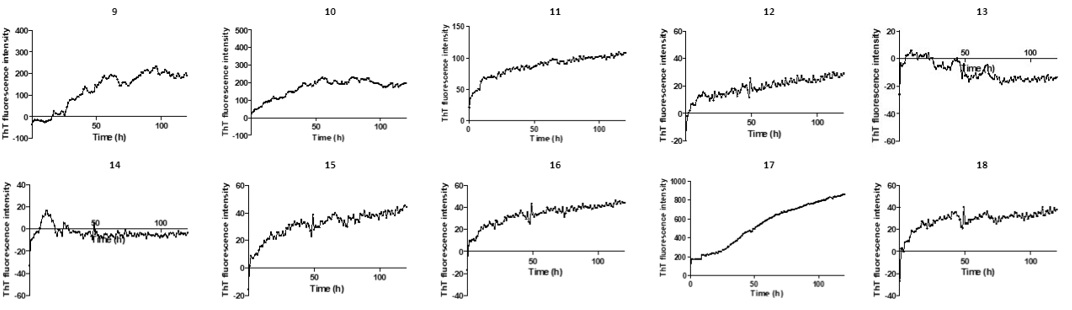


**Supplemental Figure 2.** Comparison of the Thioflavin T (ThT) fluorescence intensity. Graphs represent each species’ fibrilization intensity over 120 hours (five days) in incubation at 37 °C. Average represents experimental triplicate. Fluorescence background signal was subtracted. ID# 9: chicken; ID# 10: canine; ID# 11: Amur tiger; ID# 12: Zebra finch; ID# 13: budgerigar; ID# 14: turkey and Japanese quail; ID# 15: rainbow trout; ID# 16: Chinese soft-shelled turtle; ID# 17: mallard; ID# 18: Tasmanian dev


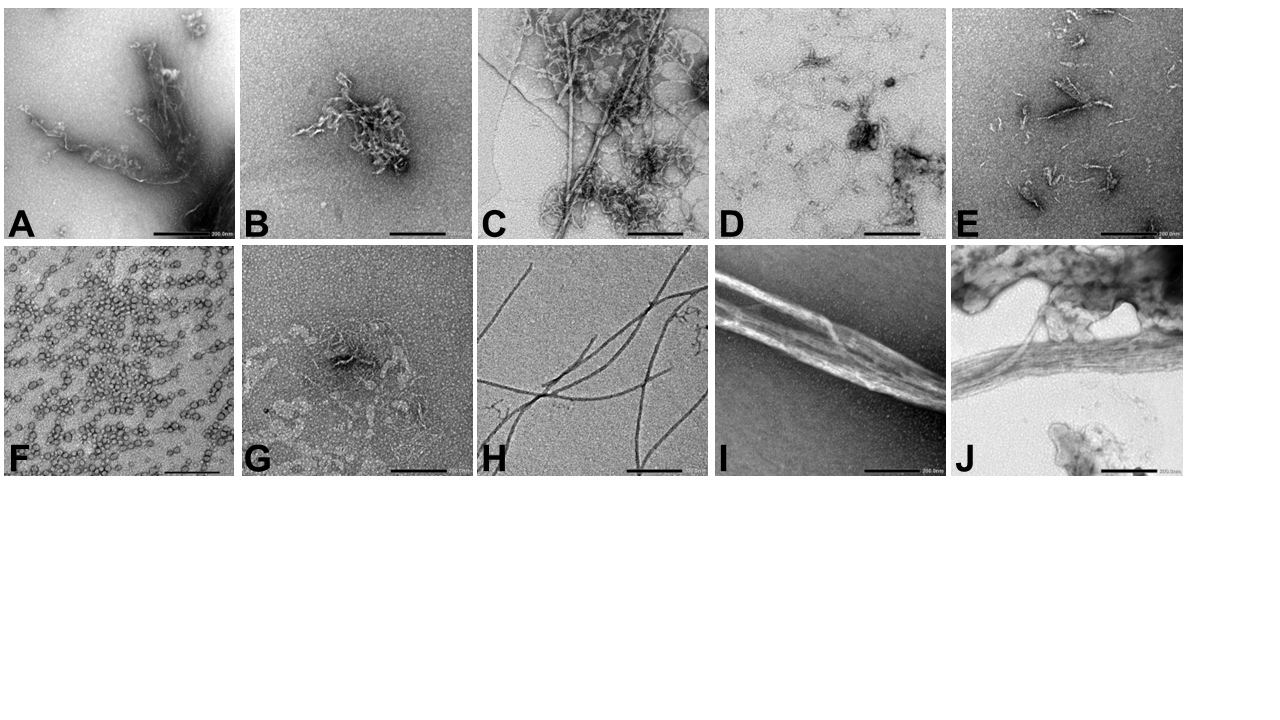


**Supplemental Figure 3.** Transmission electron microscopy (TEM) was used to observe the signal peptide, which was solubilized at 500 µM in 25 mM Tris buffer (pH 8) and 50% hexafluoroisopropanol (HFIP), and subsequently incubated at 37 °C for seven days, at a magnification of 40K. Notation A corresponding to SAA1 peptide ID# 9 (chicken). Notation B corresponding to SAA1 peptide ID# 10 (canine). Notation C corresponding to SAA1 peptide ID# 11 (Amur tiger). Notation D corresponding to SAA1 peptide ID# 12 (Zebra finch). Notation E corresponding to SAA1 peptide ID# 13 (budgerigar). Notation F corresponding to SAA1 peptide ID# 14 (turkey and Japanese quail). Notation G corresponding to SAA1 peptide ID# 15 (rainbow trout). Notation H corresponding to SAA1 peptide ID# 16 (Chinese soft-shelled turtle). Notation I corresponding to SAA1 peptide ID# 17 (mallard). Notation J corresponding to SAA1 peptide ID# 18 (Tasmanian devil). Scale bar = 200 nm.

**Discussion**

One of our most noteworthy findings was the generation of amyloid-like fibrils with the Chinese soft-shelled turtle peptide (ID# 16) by ThT and TEM, as characterization of amyloidosis in reptiles is rare in the literature (Penticoff et al. 2021). Despite the presence of fibrils, the TANGO aggregation score was only 3 for the Chinese soft-shelled turtle. The turkey, which also exhibited a low TANGO aggregation score, shares alterations with the turtle at both positions 8 and 9 aa. Unless coincidental, the shared point mutation at the same two locations could be the culprit behind the low aggregation scores of these species. Such similarities are supported by Zhou et al, who identified the Chinese soft-shelled turtle SAA sequence to be 74% identical to the duck, emphasizing that the turtle SAA sequence corresponds most to avian SAA sequences (Zhou et al. 2011). Peptide corresponding to the mallard (wild duck, ID# 17) resulted in amyloid-like fibrils with the highest ThT intensity.

As mentioned previously, the turkey/Japanese quail peptide (ID# 14) was one of the two samples that did not depict evidence of fibril formation in ThT, and its TANGO aggregation score was zero. However, when the sample was viewed via TEM, spherical structures were noted, signifying possible oligomeric species. Amyloidosis has been reported in the Japanese quail (Nakamura et al. 1998; Nakayama et al. 2017), a species whose 1-25 aa SAA sequence is identical to the turkey. In one particular study from Nakamura et al., necropsies were performed on a sample of Japanese quail from a large farm (Nakamura et al. 1998). Amyloid deposits were of greatest abundance in the liver, spleen, and gastrointestinal tracts of necropsied quail, different from the typical kidney deposits in mammals. Amyloidosis manifested as anorexia, diarrhea, and a decrease in egg production in this flock (Nakamura et al. 1998). Systemic amyloidosis has been found in domestic poultry and birds maintained in captivity (i.e. zoo and parks) (Cowan 1968; Shientag et al. 2016), with cases reported in the turkey (*Meleagris gallopavo*) (Breuer et al. 2014), but additional studies are required to confirm protein precursor and identify any predisposing factors.

The other sample resulting in a negative ThT result (flat curve) corresponded to the budgerigar (ID# 13), which were not consistent with TEM analysis. Small tiny fibrils were observed on the copper grid by TEM.

**References**

Breuer W, Moser H, De Souza-Pilz M, et al. (2014) [Amyloidosis in turkeys (Meleagris gallopavo f. domestica)--a case report]. Berl Munch Tierarztl Wochenschr 127(5-6):227-32

Cowan DF (1968) Avian amyloidosis. I. General incidence in zoo birds. Pathol Vet 5(1):51-8

DiBartola SP, Tarr MJ, Webb DM, Giger U (1990) Familial renal amyloidosis in Chinese Shar Pei dogs. J Am Vet Med Assoc 197(4):483-7

Murakami T, Inoshima Y, Sakamoto E, et al. (2013) AA amyloidosis in vaccinated growing chickens. J Comp Pathol 149(2-3):291-7 doi:10.1016/j.jcpa.2013.02.002

Nakamura K, Tanaka H, Kodama Y, Kubo M, Shibahara T (1998) Systemic amyloidosis in laying Japanese quail. Avian Dis 42(1):209-14

Nakayama Y, Kamiie J, Watanabe G, Suzuki K, Murakami T (2017) Spontaneous, Experimentally Induced, and Transmissible AA Amyloidosis in Japanese Quail ( Coturnix japonica). Vet Pathol 54(6):912-921 doi:10.1177/0300985817723692

Nemeth NM, Gonzalez-Astudillo V, Oesterle PT, Howerth EW (2016) A 5-Year Retrospective Review of Avian Diseases Diagnosed at the Department of Pathology, University of Georgia. J Comp Pathol 155(2-3):105-120 doi:10.1016/j.jcpa.2016.05.006

Penticoff HB, Hipkiss HK, Hetak AA, Agnew DW, Fortin JS (2021) Survey of amyloidosis cases among different free-living wild and zoo animals. Amyloid 28(3):145-152 doi:10.1080/13506129.2021.1940931

Schulze C, Brugmann M, Boer M, Brandt HP, Pohlenz J, Linke RP (1998) Generalized AA-amyloidosis in Siberian tigers (Panthera tigris altaica) with predominant renal medullary amyloid deposition. Vet Pathol 35(1):70-4 doi:10.1177/030098589803500108

Shientag LJ, Cabrera OA, Pazour GJ (2019) Allelic Diversity in the Serum Amyloid A2 Gene and Amyloid A Amyloidosis in a Breeding Colony of Zebra Finches (Taeniopygia guttata). Comp Med 69(5):425-431 doi:10.30802/AALAS-CM-18-000139

Shientag LJ, Garlick DS, Galati E (2016) Amyloidosis in a Captive Zebra Finch (Taeniopygia guttata) Research Colony. Comp Med 66(3):225-34

Woo S-H, Kim YA, Kwon SW, et al. (2017) Amyloidosis in a Whooper swan (Cygnus cygnus). Korean Journal of Veterinary Research 57(4):257-260 doi:10.14405/kjvr.2017.57.4.257

Zhou X, Wang L, Feng H, Guo Q, Dai H (2011) Acute phase response in Chinese soft-shelled turtle (Trionyx sinensis) with Aeromonas hydrophila infection. Dev Comp Immunol 35(4):441-51 doi:10.1016/j.dci.2010.11.011
